# Distinct fibrin clot characteristics in individuals with severe obesity and metabolic liver disease: 2-year follow-up after bariatric surgery

**DOI:** 10.1016/j.rpth.2026.103354

**Published:** 2026-01-13

**Authors:** Nadja Bødker Pedersen, Anna-Marie Bloch Münster, Mette Munk Lauridsen, Matthew James Flick, Charlotte Wilhelmina Wernberg, Elise Jonasson, Lene Rud Tarp, Niels Korsgaard, Moniek P.M. de Maat, Else-Marie Bladbjerg

**Affiliations:** 1 Unit for Thrombosis Research, Department of Clinical Biochemistry, University Hospital of Southern Denmark, Esbjerg, Denmark; 2 Department of Regional Health Research, University of Southern Denmark, Odense, Denmark; 3 Department of Gastroenterology and Hepatology, Liver Research Group, University Hospital of Southern Denmark, Esbjerg, Denmark; 4 Department of Pathology and Laboratory Medicine, UNC Blood Research Center, University of North Carolina at Chapel Hill, Chapel Hill, North Carolina, USA; 5 Department of Pathology, University Hospital of Southern Denmark, Esbjerg, Denmark; 6 Department of Hematology, Cardiovascular Institute, Erasmus University Medical Center Rotterdam, Rotterdam, the Netherlands

**Keywords:** fatty liver, fibrin, fibrosis, inflammation, obesity

## Abstract

**Background:**

Obesity predisposes individuals to metabolic dysfunction–associated steatotic liver disease (MASLD), the hepatic manifestation of metabolic syndrome. Studies in mice suggest that fibrin deposition in adipose tissue and liver promotes obesity and MASLD, but whether similar mechanisms are linked to metabolic disease in humans is incompletely studied.

**Objectives:**

This study determined the relationship between plasma fibrin clot characteristics and hepatic fibrin deposition with obesity and MASLD severity and examined changes in fibrin measures 2 years after bariatric surgery.

**Methods:**

We included 195 individuals with body mass index (BMI) of > 35 kg/m^2^ in a cohort study. A subgroup of 93 individuals who underwent bariatric surgery (*n* = 35) or served as nonsurgical controls (*n* = 58) was followed for 2 years. Clot characteristics were studied by turbidity. Hepatic tissue samples were scored for MASLD, and in 3 individuals with varying BMI, tissues were stained for fibrin.

**Results:**

Examination of plasma clots revealed that individuals with BMI > 45 kg/m^2^ had lower clot lysis and fiber density, while maximal turbidity increment (*V*_max_), fiber diameter, and maximum absorbance (MA) were higher than those in individuals with BMI < 40 kg/m^2^. *V*_max_, fiber density, and MA were lower in individuals with metabolic dysfunction–associated steatohepatitis than those in patients without MASLD. After bariatric surgery, clot lysis and fiber density were higher than that in controls, whereas *V*_max_, fiber diameter, and MA were lower. Staining intensity of hepatic fibrin deposits increased with obesity severity but was not clearly reduced postsurgery.

**Conclusion:**

Severe obesity and MASLD are associated with altered fibrin characteristics in plasma clots and liver tissue, suggesting fibrin(ogen) reflects metabolic alterations although the directionality remains to be clarified.

## Essentials

• Fibrin formation promotes severe obesity and metabolic liver disease in mice.
• We studied fibrin formation in patients with severe obesity before and after bariatric surgery.
• Severe obesity was linked to increased plasma and hepatic tissue fibrin formation.
• Metabolic liver disease was linked to decreased plasma fibrin formation.

## 1 Introduction

One in 8 people worldwide is living with obesity [1], and these individuals have a 3.5-fold increased risk of developing metabolic dysfunction–associated steatotic liver disease (MASLD) [2]. MASLD is a spectrum disorder initiated by excessive lipid accumulation in hepatocytes that provokes inflammation, fibrosis, and compromised liver function. Indeed, MASLD ranges from simple steatosis to metabolic dysfunction–associated steatohepatitis (MASH), which can progress into irreversible fibrosis and cirrhosis or hepatocellular carcinoma [3].

A recent study in mice [4] linked the central protein of the coagulation cascade, fibrinogen, to obesity and liver steatosis based on the ability of fibrin(ogen) to interact with leukocytes, thus provoking the chronic inflammation that exacerbates metabolic dysfunction in adipose and liver tissue [[5], [6], [7]]. Kopec et al. [4] demonstrated that transgenic mice with a mutant form of fibrinogen (Fiby^390-396A^, unable to bind to leukocytes) were protected against diet-induced obesity and MASLD and that less fibrin(ogen) depositions were found in the adipose and hepatic tissues of these mice compared with those in mice with normal fibrinogen. They supported their findings by demonstrating increased hepatic fibrin(ogen) depositions in pediatric patients with MASH (*n* = 6) [4]. These observations lead to the notion that fibrin fuels fatty liver disease [8] based on the implied involvement of fibrinogen and fibrin in the development of obesity and MASLD. It is, however, unknown how fibrin formation is linked to obesity and MASLD in humans.

Fibrin formation occurs when thrombin cleaves fibrinopeptide A and B from fibrinogen, initiating the polymerization of fibrin monomers and leading to the generation of a 3-dimensional fibrin network [9]. Fibrin(ogen) depositions can be detected in tissues, such as liver biopsies, with immunohistochemical staining techniques using monoclonal [10] or polyclonal [4] fibrinogen or fibrin antibodies [11]. In plasma, the potential of fibrinogen to form fibrin can be studied by turbidity, analyzing characteristics of fibrin fiber polymerization, structure of the fibrin clot, and the subsequent clot lysis [12,13]. Several studies have shown that not only levels of total fibrinogen influence plasma fibrin clot formation, as reviewed by Undas and Ariëns [14], but also the naturally occurring fibrinogen variants, fibrinogen γ' [15], fibrinogen α_E_ [16], and sialylated fibrinogen [17,18] are known to produce thinner fibers and a more dense fibrin network [15,16,19].

We have recently shown that human plasma levels of these 3 fibrinogen variants are associated with the severity of obesity and MASLD, suggesting that the variants play a role in these inflammatory conditions [20]. In this study, we investigated an association between fibrin formation and the severity of obesity and steatotic liver disease. Specifically, we analyzed whether plasma fibrin clot characteristics and hepatic fibrin deposition are associated with the severity of obesity and MASLD in a large cohort of individuals with body mass index (BMI) > 35 kg/m^2^ (substudy 1) and whether these variables change 2 years after bariatric surgery (substudy 2). We hypothesized that the severity of obesity and MASLD associates with plasma fibrin clot characteristics and hepatic tissue fibrin depositions in the direction of increased fibrin formation and that fibrin formation is reduced 2 years post-surgery.

## 2 Materials and Methods

### 2.1 Participants and study design

This study is a part of the ongoing single-center prospective parallel controlled study, PROMETHEUS, at the University Hospital of Southern Denmark, Esbjerg, Denmark. The PROMETHEUS study includes individuals aged 18 to 70 years with a BMI of > 35 kg/m^2^, and half of the participants are undergoing bariatric surgery as described in detail by Indira Chandran et al. [21]. All participants gave written informed consent. The study was conducted according to the Declaration of Helsinki, and the project was approved by the Regional Ethics Committee of Southern Denmark (DK S-10160006G) and registered at ClinicalTrial.gov (NCT03535142).

**Figure 1:**
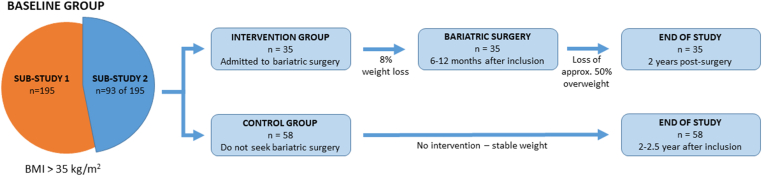
Study design. Substudy 1 included 195 participants examined at baseline, and substudy 2 included a subgroup of 93 participants, who were assigned to either intervention (bariatric surgery) or a control group. Participants in substudy 2 were also examined 2 years post-surgery (intervention group) or approximately 2.5 years after baseline examination (control group). BMI, body mass index. Previously published by Pedersen et al. [20].

The participants and study design of the present study were described in detail elsewhere [20]. In brief, we included 195 individuals between 2018 and 2022, who were recruited based on national criteria for bariatric surgery referral (BMI > 35 kg/m^2^ with obesity-related comorbidities or BMI > 50 kg/m^2^ with obesity-related social or physical complications), and they were all examined at baseline (substudy 1). A subgroup of 93 participants either underwent bariatric surgery (Roux-en-Y gastric bypass [*n* = 28] or gastric sleeve [*n* = 7]) or served as a nonsurgical control group (*n* = 58) and were followed 2 years post-surgery (bariatric group) or approximately 2.5 years from baseline examination (control group). At both visits (baseline and end of study) anthropometric measurements were included, and blood samples and liver biopsies were collected. The study design is presented in Figure 1. We excluded individuals in both substudies with an alcohol intake >7 and 14 units/wk for women and men, respectively; chronic liver diseases other than MASLD; use of hepatotoxic drugs (eg, tamoxifen, methotrexate, and glucocorticoids); decompensated cirrhosis; pregnancy; severe illness; low carbohydrate diets; use of antibiotics within the last 2 months; and use of all types of anticoagulant therapy within the previous 6 months. Table 1 presents the baseline characteristics of participants in substudy 1. Supplementary Tables 1–4 present previously published characteristics of the participants stratified on BMI, MASLD, and fibrosis (substudy 1) and before and after bariatric surgery (substudy 2) [20]. We also included a normal-weight reference group consisting of 120 healthy blood donors (60 men and 60 women, mean age of 30 years, mean BMI of 25.6 kg/m^2^, no use of oral contraceptives and medicine).

**Table 1:** Clinical characteristics at baseline for participants in substudy 1.

Variable	Substudy 1 (*n* = 195)
Sex: women	141 (72)
Age (y)	44.2 (42.4-45.9)
Ethnicity	
White	190 (97)
Black	1 (0.5)
Latino	2 (1)
Other	2 (1)
Smokers	27 (14)
Weight (kg)	124 [108-138]
BMI (kg/m^2^)	43.1 (42.2-44.1)
Waist-hip ratio	0.88 [0.82-0.98]
SAF score	
No MASLD	57 (29)
MASLD	95 (49)
MASH	43 (22)
Fibrosis grade	
No fibrosis	48 (25)
Mild fibrosis	91 (47)
Significant fibrosis	57 (29)
ALT (U/L)	32 (22-53)
AST (U/L)	25 (21-35)
Creatinine (μmol/L)	66 (59-75)
Total cholesterol (mmol/L)	4.5 (4.4-4.7)
HDL cholesterol (mmol/L)	1.2 (1.0-1.3)
LDL cholesterol (mmol/L)	3.0 (2.9-3.2)
HbA1c (mmol/mol)	37 (34-42)
Medication	
Antihypertensives	84 (43)
Antidiabetics	47 (24)
Metformin	37 (19)
GLP-1 analogs	17 (9)
SGLT-2 inhibitors	11 (6)
Insulin	06 (3)
Others^a^	03 (2)
Statins	51 (26)
Antidepressants	67 (34)
Oral contraceptives	12 (6)

Data are presented as mean (95% CI), median (IQR), or *n* (%).

BMI, body mass index; SAF, steatosis, activity, and fibrosis; MASLD, metabolic dysfunction–associated steatotic liver disease; MASH, metabolic dysfunction–associated steatohepatitis; ALT, alanine transaminase; AST, aspartate transaminase; HDL, high-density lipoprotein; LDL, low-density lipoprotein; HbA1c, hemoglobin A1c; GLP-1, glucagon-like peptide-1; SGLT-2, sodium-glucose cotransporter.

a Others include dipeptidyl peptidase IV inhibitors and sulfonylurea drugs. Previously published by Pedersen et al. [20].

### 2.2 Blood sampling

Venous blood samples were drawn in the morning after an overnight fast and 15 minutes of rest in a supine position. Samples were collected in citrate tubes (Becton, Dickinson & Co; reference: 363048) and centrifuged at 2000 × *g* (20 °C) for 20 minutes to obtain platelet-poor plasma before being stored in aliquots at −80 °C until analysis.

### 2.3 Plasma analyses

Analytical methods used for previously reported variables (Supplementary Tables 1–4) are described elsewhere [20]. Fibrin polymerization and lysability were determined using turbidity measurements as described by Sjøland et al. [13] with a few adjustments. In brief, fibrin polymerization was initiated by mixing plasma with thrombin (1 IU/mL, final concentration) and CaCl_2_ (15 mmol/L, final concentration) in both the presence and absence of recombinant tissue plasminogen activator (300 ng/mL, final concentration). Turbidity was observed for 30 minutes at 340 nm on a Sunrise absorbance microplate reader (Tecan Group Ltd), and the maximal turbidity increment (*V*_max_), maximum absorbance (MA), and percentage of fibrin clot lysis were calculated. The plate was sealed and incubated overnight at room temperature, and the following day, the variables of fibrin clot structure (fiber diameter and fiber mass density) were calculated based on optical density (OD) measurements at 340, 405, 540, 608, and 690 nm according to previous studies [12,22]. Interassay coefficients of variation for the fibrin clot characteristics were 12% for *V*_max_, 3% for MA, 15% for clot lysis, and 7% for fiber diameter and fiber mass density.

### 2.4 Histopathology

Liver biopsies were collected using a suction needle and reviewed by a single pathologist blinded to other data as described elsewhere [20]. In brief, the severity and progression of MASLD were evaluated using the steatosis, activity, and fibrosis (SAF) score: no MASLD, MASLD, and MASH. The SAF score assesses steatosis (S, S0-S3), lobular inflammation (A, A0-A2), and ballooning (A, A0-A2). MASLD is defined by the presence of steatosis in >5% of hepatocytes, while MASH is characterized by steatosis grades 1 to 3, hepatocellular ballooning, and any degree of lobular inflammation [23]. Finally, the fibrosis grade (F, F0-F4) was determined using the Kleiner fibrosis score, with F4 indicating cirrhosis [24].

### 2.5 Fibrin immunohistochemistry

In a small pilot study, liver tissue samples from 3 participants at baseline and 2 years after surgery were embedded in paraffin and sectioned (4 μm). These 3 participants were selected to obtain a large variation in BMI at baseline. The sections were unmasked with a Proteinase K solution, followed by immunohistochemical staining using an automated staining machine (Benchmark ULTRA; Roche Diagnostics). A mouse monoclonal antihuman fibrin antibody, clone 59D8 (Merck), was used for the staining. This antibody is specific toward human fibrin and does not crossreact with fibrinogen [25]. For detection, a commercial detection kit (UltraView Universal Alkaline Phosphatase Red Detection Kit; Roche Diagnostics) was used.

Whole-slide images of liver biopsies were acquired using a digital pathology system (Sectra). Quantification of fibrin-positive staining was performed using QuPath (version 0.5.1 for Windows; University of Edinburgh, United Kingdom). For each slide, the entire liver tissue section was manually annotated as the region of interest. A custom Fast Red channel was defined using a positively stained area in which a pixel classifier was applied using identical smoothing sigma and threshold parameters across all images. The percentage of fibrin-positive area (% area) was calculated based on the proportion of positively stained pixels within the region of interest.

### 2.7 Statistical analysis

The sample size calculation was based on total plasma fibrinogen as described elsewhere [20] and resulted in a total of 180 participants in substudy 1 and 2 × 60 participants in substudy 2. In substudy 1, we stratified participants into 3 groups by severity of overweight based on BMI (BMI < 40 kg/m^2^, BMI = 40-45 kg/m^2^, and BMI > 45 kg/m^2^), by severity of MASLD based on the SAF score (no MASLD, MASLD, and MASH), and by severity of fibrosis based on Kleiner fibrosis score (no fibrosis [F0], mild fibrosis [F1], and clinically significant fibrosis [F2-F4]). These stratifications were performed independently and were not internally controlled for each other. Therefore, results for fibrin clot properties within these groups may be influenced by overlapping characteristics, such as severe obesity in MASLD/MASH or fibrosis groups. Plasma fibrin clot characteristics were compared between groups using linear regression analysis and adjusted for the confounders—sex and age—chosen a priori. Here, “sex” denotes biological sex assigned at birth. Group comparisons were adjusted only for age and sex to avoid overadjustment for potential mediators (eg, fibrinogen concentration, inflammation, and diabetes), but residual confounding cannot be excluded. Additional sensitivity analyses including diabetes medication, statin use, and total fibrinogen were performed and did not change the overall findings. Plasma fibrin clot characteristics were compared between healthy blood donors and BMI, MASLD, and fibrosis groups using analysis of variance (anova) or Kruskal–Wallis test. Pairwise comparison of groups was performed using Tukey honestly significant difference test or Dunn multiple comparisons test if the initial test was significant.

In substudy 2, a paired *t*-test or a Wilcoxon signed-rank test was used as appropriate for within-group comparisons (between baseline and follow-up in surgery and control groups) of plasma fibrin clot characteristics. Fibrin clot characteristics were compared between the surgery and control groups after the follow-up period using linear regression analysis adjusted for baseline values and minor differences in BMI [20]. Associations of changes in body weight and BMI with changes in fibrin clot characteristics were estimated using Spearman rank correlation coefficient (*r*_s_).

Results are presented as mean (95% CI or SD) or median (IQR) as appropriate for continuous variables and as percentages for categorical variables. A *P* value of < .05 was considered statistically significant. All statistical analyses were performed using STATA-18 SE (StataCorp LLC), and figures were made in GraphPad Prism version 10 (GraphPad Software).

## 3 Results

### 3.1 Substudy 1

**Figure 2:**
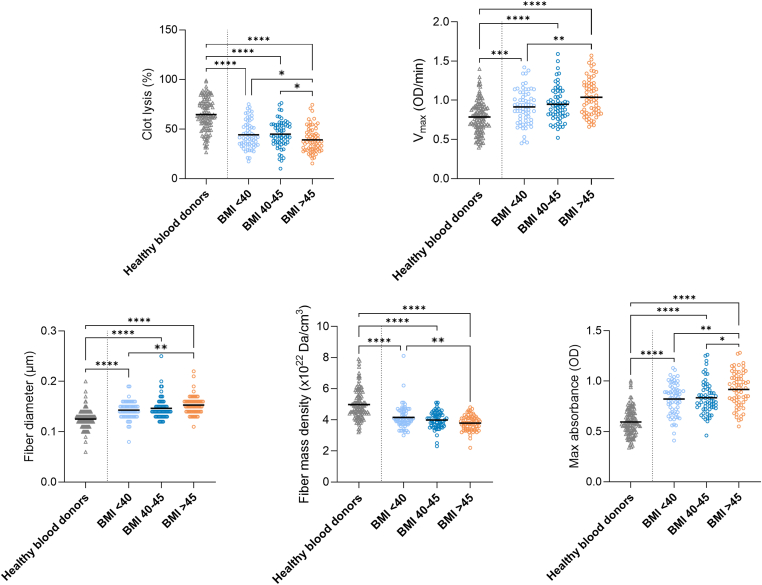
Data for fibrin clot characteristics in healthy controls (mean BMI, 25.6 kg/m^2^; *n* = 120) and BMI groups of severe obesity (BMI < 40 kg/m^2^, *n* = 65; BMI = 40-45 kg/m^2^, *n* = 64; BMI > 45 kg/m^2^, *n* = 66) are presented as mean or median (fiber diameter and fiber density). BMI groups were compared using linear regression analysis adjusted for age and sex, and healthy controls and BMI groups were compared using anova or Kruskal–Wallis test (fiber diameter and fiber density). BMI, body mass index; OD, optical density; *V*_max_, maximal turbidity increment. ∗*P* < .05; ∗∗*P* < .01; ∗∗∗*P* < .001; ∗∗∗∗*P* < .0001.

In substudy 1, individuals in the BMI > 45 kg/m^2^ group had reduced fibrin clot lysis and increased MA compared with those in the BMI 40-45 kg/m^2^ (Δclot lysis, −5.77%; 95% CI, −11.44% to −0.10%; ΔMA, 0.08 OD; 95% CI, 0.01-0.15 OD) and BMI < 40 kg/m^2^ groups (Δclot lysis: −5.95%; 95% CI, −11.71 to −0.20%; ΔMA: 0.10 OD; 95% CI, 0.03-0.17 OD). *V*_max_ (Δ, 0.11 OD/min; 95% CI, 0.01-0.20 OD/min) and fiber diameter (Δ, 0.01 μm; 95% CI, 0.00-0.02 μm) were higher in individuals with the highest BMI than those in individuals with the lowest BMI, while the opposite was observed for fiber density (Δ, −0.39 × 10^22^ Da/cm^3^; 95% CI, −0.65 to −0.13 × 10^22^ Da/cm^3^) (Figure 2).

**Figure 3:**
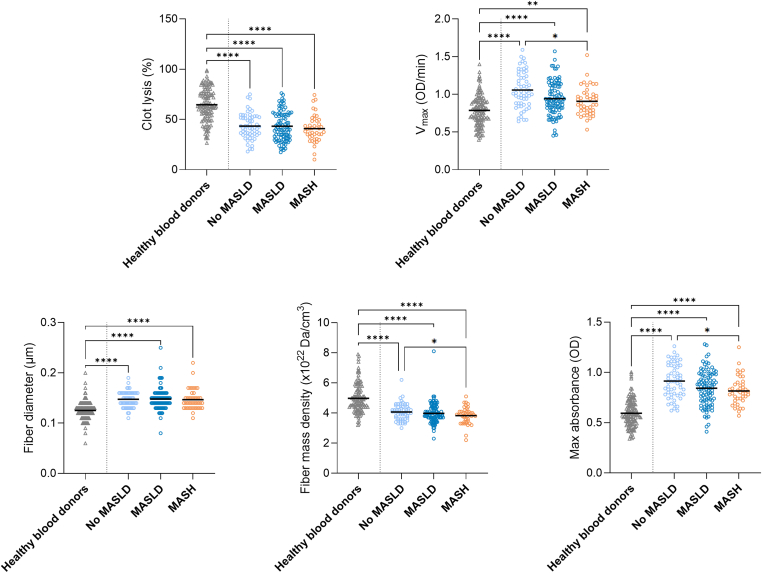
Data for fibrin clot characteristics in healthy controls (mean BMI, 25.6 kg/m^2^; *n* = 120) and MASLD groups (no MASLD, *n* = 55; MASLD, *n* = 96; MASH, *n* = 44) are presented as mean or median (fiber diameter and fiber density). MASLD groups were compared using linear regression analysis adjusted for age and sex, and healthy controls and MASLD groups were compared using anova or Kruskal–Wallis test (fiber diameter and fiber density). MASH, metabolic dysfunction–associated steatohepatitis; MASLD, metabolic dysfunction–associated steatotic liver disease; OD, optical density; *V*_max_, maximal turbidity increment. ∗*P* < .05; ∗∗*P* < .01; ∗∗∗∗*P* < .0001.

**Figure 4:**
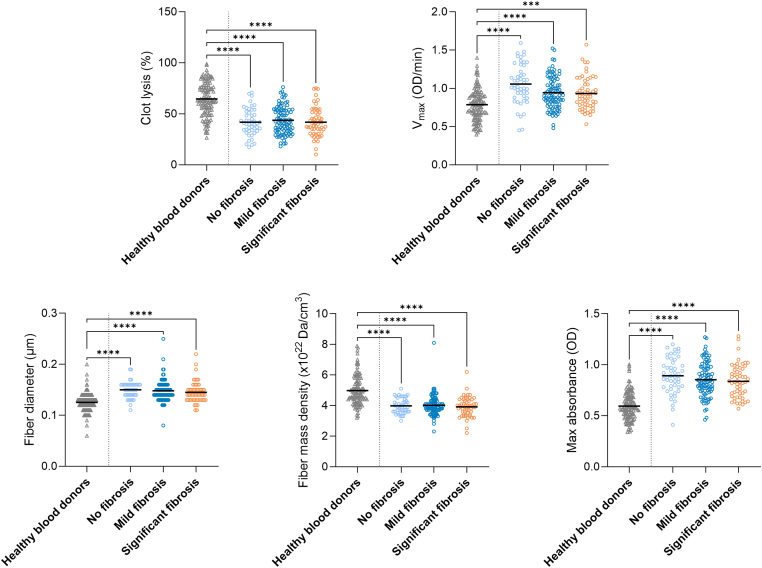
Data for fibrin clot characteristics in healthy controls (mean BMI, 25.6 kg/m^2^; *n* = 120) and fibrosis groups (no fibrosis, *n* = 47; mild fibrosis, *n* = 91; clinically significant fibrosis, *n* = 57) are presented as mean or median (fiber diameter and fiber density). Fibrosis groups were compared using linear regression analysis adjusted for age and sex, and healthy controls and fibrosis groups were compared using anova or Kruskal–Wallis test (fiber diameter and fiber density). OD, optical density; *V*_max_, maximal turbidity increment. ∗∗∗*P* < .001; ∗∗∗∗*P* < .0001.

Fibrin clot characteristics for the MASLD groups are presented in Figure 3. *V*_max_ (Δ, −0.13 OD/min; 95% CI, −0.23 to −0.02 OD/min), fiber density (Δ, −0.31× 10^22^ Da/cm^3^; 95% CI, −0.60 to −0.15 × 10^22^ Da/cm^3^), and MA (Δ, −0.09 OD; 95% CI,−0.17 to −0.01 OD) were lower in the group with MASH than those in the group without MASLD. There were no differences between the groups concerning percent clot lysis or fiber diameter. When comparing the participants based on severity of fibrosis, no significant differences were observed between the groups (Figure 4).

Figure 2, Figure 3, Figure 4 also present fibrin clot characteristics of BMI, MASLD, and fibrosis groups compared with healthy blood donors. Across all stratifications, clot lysis and fiber density were consistently lower in participants with obesity, while *V*_max_, fiber diameter, and MA were higher. These differences were statistically significant for all comparisons with healthy blood donors (*P* < .001 for most variables), whereas internal differences between patient subgroups were generally smaller.

### 3.2 Substudy 2

**Figure 5:**
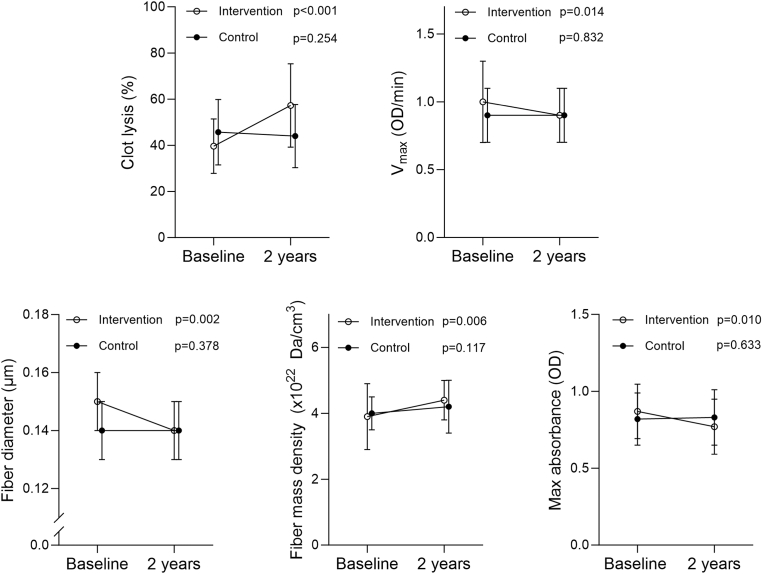
Data for fibrin clot characteristics in the intervention (bariatric surgery, *n* = 35) and control (nonsurgical, *n* = 58) groups are presented as mean and SD or median and 75th percentile (fiber diameter). Baseline and 2-year follow-up data were compared with a paired *t*-test or Wilcoxon signed-rank test (fiber diameter) within the groups. OD, optical density; *V*_max_, maximal turbidity increment.

In substudy 2, clot lysis and fiber density increased, while *V*_max_, fiber diameter, and MA decreased 2 years after bariatric surgery when compared with the levels before surgery (Figure 5). There were no changes in the control group. At the end of study, clot lysis (Δ, 18.79%; 95% CI, 13.69%-23.89%; *P* < .001) and fiber density (Δ, 0.27 × 10^22^ Da/cm^3^; 95% CI, −0.02 to 0.57 × 10^22^ Da/cm^3^; *P* = .066) were higher in the intervention group than those in the control group, and V_max_ (Δ, −0.08 OD/min; 95% CI, −0.16 to 0.00 OD/min; *P* = .054), fiber diameter (Δ, −0.01 μm; 95% CI, −0.01 to 0.00 μm; *P* = .054), and MA (Δ, 0.09 OD; 95% CI, −0.16 to −0.03 OD; *P* = .007) were lower.

Table 2 presents correlations of reductions in body weight and BMI with changes in fibrin clot characteristics in the surgery group, showing negative associations of weight loss with clot lysis and fiber density (*r*_s_, −0.41 to −0.48), while there were positive associations for *V*_max_, fiber diameter, and MA (r_s_, 0.30-0.51).

**Table 2:** Correlations between changes in body weight and BMI and changes in fibrin clot characteristics before and after bariatric surgery (*n* = 35).

Variable	ΔBody weight (kg)		ΔBMI (kg/m^2^)	
	*r*_s_	*P*	*r*_s_	*P*
ΔClot lysis (%)	−0.42	.01	−0.42	.01
Δ*V*_max_ (OD/min)	0.42	.01	0.51	.002
ΔFiber diameter (μm)	0.30	.08	0.36	.03
ΔFiber density (×10^22^ Da/cm^3^)	−0.41	.02	−0.48	.004
ΔMaximum absorbance (OD)	0.47	.005	0.51	.002

BMI, body mass index; OD, optical density; *r*_s_, Spearman correlation coefficient; *V*_max_, maximal turbidity increment.

### 3.3 Hepatic fibrin deposition

**Figure 6:**
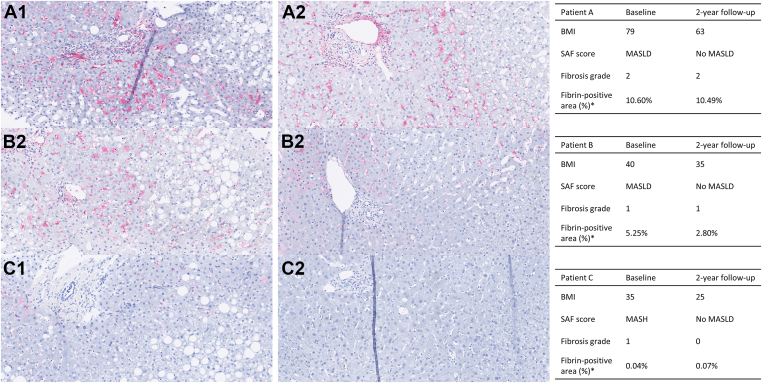
Immunohistochemical stainings for fibrin (red) of liver tissue from 3 patients with severe obesity at baseline (A1–C1) and 2 years after bariatric surgery (A2–C2). All images show histologically comparable regions, including the portal triad and surrounding zones, selected in collaboration with a pathologist to ensure consistency across samples. Artifactual vertical lines seen in A1, B2, and C2 are due to minor tissue folding during sample preparation. ∗Fibrin-positive area (%) refers to the entire whole-slide image, not the magnified region shown. Magnification, 40×. BMI, body mass index; MASH, metabolic dysfunction–associated steatohepatitis; MASLD, metabolic dysfunction–associated steatotic liver disease; SAF, steatosis, activity, and fibrosis.

To investigate a possible connection between fibrin formation in hepatic tissues and the severity of obesity and MASLD, we analyzed fibrin accumulation by immunohistochemistry in liver tissues from 3 patients before and 2 years after bariatric surgery. Figure 6 presents representative staining areas for the 3 patients. The intensity of fibrin staining was stronger at high BMI, but there were no clear changes in fibrin staining after weight loss (and disappearance of MASLD) across the 3 individuals, most likely due to large variations in the degree of steatosis, MASLD, and fibrosis in the samples. Almost no fibrin deposition was observed in the steatotic areas, but fibrin was located in the area of the portal triad, the periportal zone, the sinusoids, and in Disse space between the sinusoids and the hepatocytes. Inflammation and fibrosis were observed around the portal triad, whereas steatosis was seen in zone 3 closest to the central vein.

## 4 Discussion

We hypothesized that plasma fibrin clot characteristics and hepatic tissue fibrin formation associate with the severity of obesity and MASLD in the direction of increased fibrin formation and that fibrin formation is reduced 2 years after bariatric surgery. We demonstrated increased plasma fibrin formation and reduced fibrin clot lysis in individuals with the most severe obesity. The fibrin fibers were thicker and with a lower density. Hepatic tissues from 3 individuals also showed more intense fibrin staining with increased BMI. After bariatric surgery, clots tended to form slower, be more susceptible to lysis, and the fibrin fibers were thinner and structurally denser. In individuals with more advanced MASLD, clot characteristics shifted toward reduced fibrin formation and fiber thickness. These results are summarized in Supplementary Figure 1. No consistent differences were observed between fibrosis stages. Interpretation of hepatic staining patterns in relation to obesity and MASLD was limited by the number of available tissue samples.

We found that clots formed in individuals with higher BMI or waist-hip ratio (results not shown) are more prothrombotic, as indicated by an increase in *V*_max_, fiber diameter, and MA and a decrease in clot lysis in the group with the highest BMI. These observations align with the observed changes in clot characteristics 2 years after bariatric surgery: a decrease in *V*_max_, fiber diameter, and MA and an increase in clot lysis. These results are also consistent with observations from Stolberg et al. [26], reporting similar findings on fibrin clot characteristics 6 months after bariatric surgery. However, a study in 32 patients with severe obesity also found reductions in MA, but no change in clot lysis 9 months postsurgery [27]. Other studies have shown that increasing BMI is associated with prolonged clot lysis time [[28], [29], [30]]. Building on this, our data further suggest that individuals with very severe obesity (BMI > 45 kg/m^2^) may represent a distinct subgroup with more prothrombotic clot characteristics compared with those with moderate obesity, indicating a potentially elevated thrombotic risk. These findings are further underscored by the pronounced differences observed between participants with severe obesity and healthy blood donors, where clots in healthy donors consistently exhibited less prothrombotic characteristics across all variables (Figure 2). Since compact and rigid clots are more resistant to lysis and are observed in patients with or at risk of cardiovascular disease [14], our results suggest (except for the increase in fiber density) that the metabolic restoration associated with bariatric surgery improves the fibrin clot characteristics, making the clots less prothrombotic. Interestingly, it remains unclear whether a less prothrombotic fibrin clot can be translated into a less proinflammatory fibrin network with a reduced potential to attract leukocytes and thereby promote inflammation.

The associations between severe obesity and increased formation of prothrombotic clots are further supported by the observations in healthy blood donors showing significantly less prothrombotic clots compared with the obesity cohort (Figure 2) and by the correlations between weight loss and changes in fibrin clot characteristics (Table 2). However, the mechanisms linking weight loss to antithrombotic fibrin clot properties were not clarified. Changes in clot properties might be mediated by weight loss alone, but more likely by metabolic improvements such as reductions in the proinflammatory cytokines interleukin 6 and tumor necrosis factor α, which are synthesized in adipose tissue [31]. Both interleukin 6 and tumor necrosis factor α affect the hepatic production of fibrinogen [32], a major determinant of fibrin clot characteristics [33]. In the present study, other metabolic improvements after weight loss include reductions in low-density lipoprotein cholesterol and hemoglobin A1c, but we observed no association between these favorable restorations and changes in clot properties. In addition, since antidiabetic medication is known to affect *ex vivo* clots [14], discontinuation of this medication postsurgery might also explain some of the changes observed in the fibrin clot characteristics. In general, the patient cohort used a wide range of medications, which in many cases were discontinued postsurgery (Supplementary Table 4). However, our group has previously shown that clot characteristics changed similarly in a medication-free group of 19 patients 6 months after bariatric surgery [26].

When comparing MASLD groups, clots formed in individuals with MASH appeared less prothrombotic. Specifically, clots in individuals without MASLD formed more quickly (higher *V*_max_), had a greater MA, and showed higher fiber density than those in individuals with MASH. However, no differences were observed in clot lysis or fiber diameter. Only 2 studies have been published that investigated fibrin clot properties in relation to MASLD, and these used techniques other than turbidity. In the study by Hickman et al. [34], clot lysis in whole blood was analyzed using thromboelastography and showed reduced clot lysis in patients with MASLD (*n* = 28) compared with that in normal-weight healthy controls (*n* = 22). However, the study found no differences in clot lysis between patients with MASLD when looking into increasing grades of steatosis or the presence of MASH [34], confirming observations of our study. Importantly, while our findings indicated that clots in individuals with MASH appear less prothrombotic compared with those without MASLD, all MASLD subgroups still exhibited more prothrombotic clot characteristics than healthy blood donors (Figure 3), similar to the pattern reported by Hickman et al. [34]. Potze et al. [35] observed lower clot density, analyzed by confocal microscopy, in patients with simple steatosis (*n* = 24) than that in patients with MASH (*n* = 22) and MASH cirrhosis (*n* = 22) [35]. These findings contradict the observations in our study, where we observed a lower fiber density in patients with MASH than that in those without MASLD. It might be speculated why not only fiber density but also *V*_max_ and MA decreased in an antithrombotic direction in individuals with MASH in our study. Concentrations of the liver enzymes were higher in individuals with MASH than that in those with no MASLD, but total fibrinogen levels did not differ between the groups (Supplementary Table 2). Further, hypersialylation of fibrinogen may lead to defective fibrinogen polymerization in patients with cirrhosis [36,37], but we did not observe MASLD group differences in the levels of sialylated fibrinogen [20], and patients in our study did not have cirrhosis. The most likely explanation is that the use of metformin and insulin was more common in individuals with MASH (Supplementary Table 2), and these drugs were previously reported to lower *V*_max_ and MA [38]. We therefore repeated the MASLD group comparisons in individuals without diabetes (*n* = 147), but this did not change the results. Larger studies are needed to understand how MASLD affects fibrin clot characteristics and preferably using turbidity as well as imaging techniques.

In a small pilot study, we found an increase in hepatic fibrin staining intensity with severity of obesity, supporting the previous findings by Kopec et al. [4], showing fibrin(ogen) depositions in the sinusoids in liver tissue from 6 pediatric patients with MASH. They also demonstrated no fibrin(ogen) depositions in normal-weight individuals, which is similar to the results in our study, where almost no fibrin depositions were found in patient C, who achieved a BMI of 25 kg/m^2^ after the 2-year follow-up period (Figure 6C2). In another recently published study, the authors investigated fibrin(ogen) depositions in liver biopsies from patients with different inflammatory liver diseases, including MASH [10]. In this study, they detected only fibrin(ogen) depositions in 25% of the stained liver biopsies, and moderate fibrin(ogen) expression was detected in 1 of 20 biopsies from patients with MASH. A possible explanation for the differences in detection of fibrin(ogen) depositions might be the use of different antibodies. In the study by Kopec et al. [4], they used a rabbit polyclonal antimouse fibrinogen antibody for the staining of mouse liver tissue and a rabbit polyclonal antihuman fibrinogen antibody for the staining of human liver tissue. In the study by von Meijenfeldt et al. [10], they used a mouse monoclonal antihuman fibrinogen antibody, and in the present study, we used a mouse monoclonal antihuman fibrin antibody. As discussed by von Meijenfeldt et al. [10], there could be differences in specificity between monoclonal and polyclonal antibodies. In our study, the largest difference is the use of a fibrin-specific antibody compared with the previously used fibrinogen antibodies. These divergent studies clearly show the need for larger studies using the same antibodies and the same quantification technique in order to describe the intensity of fibrin staining in relation to the severity of obesity and metabolic liver disease.

Strengths of this study are that characteristics of fibrin formation are investigated both systemically in plasma and locally in liver tissue in a cohort of individuals with obesity and histological characterization of metabolic liver disease (substudy 1). Another strength is the possibility of comparing a bariatric surgery group with a nonsurgical control group, with both groups having the same long follow-up time of 2 years (substudy 2). Study limitations are that liver biopsies were only studied in 3 patients, and larger studies are definitely needed to conclude on possible associations between tissue fibrin depositions and the severity of obesity or metabolic liver disease. Another limitation is that turbidity measurements were performed only with thrombin activation. Using tissue factor, which initiates coagulation earlier, or whole-blood methods could have provided additional insights. Therefore, interpretation of the turbidity measures as markers of *in vivo* thrombotic or inflammatory activity is limited. Other study limitations include the possibility of statistical type 2 errors. The power calculation was based on total fibrinogen [20], but we managed to include only 35 (and not 60) participants in the surgery group (substudy 2), primarily due to recruitment challenges during the COVID-19 pandemic. Despite this, analyses showed significant differences in fibrinogen between groups at end of study.

In conclusion, we demonstrated that fibrin clot characteristics are associated with severe obesity in a prothrombotic direction with increased fibrin formation. Comparisons with healthy blood donors indicated that severe obesity, irrespective of MASLD or fibrosis status, is associated with substantial alterations in fibrin clot structure and function. Based on these results, all plasma variables changed in the expected direction 2 years after bariatric surgery, showing formation of less prothrombotic plasma clots. In relation to MASLD severity, clot characteristics appeared to move in an antithrombotic direction. These observations suggest that plasma clot properties reflect metabolic alteration, although the directionality and underlying mechanisms remain to be clarified. Despite the limited sample size of 3 patients, our observations of increased tissue fibrin staining with increased BMI should prompt future studies to comprehensively assess fibrin deposition in liver and adipose tissue in a larger group of patients with obesity. Ultimately, our goal is to determine whether fibrin formation is mechanistically linked to the development of obesity and MASLD, as previously suggested in mice [4].
