# Supplementary material for: Distinct fibrin clot characteristics in individuals with severe obesity and metabolic liver disease: 2-year follow-up after bariatric surgery
Source: Res Pract Thromb Haemost. 2026 Jan 13;10(1):103354. doi: 10.1016/j.rpth.2026.103354 (PMC12892059; doi:10.1016/j.rpth.2026.103354)
Supplement: Supplementary Material [file mmc1.docx]

Table S1. Clinical characteristics of BMI groups

| Variable | BMI <40 kg/m^2^ (n=65) | BMI 40-45 kg/m^2^ (n=64) | BMI >45 kg/m^2^ (n=66) | | P | |
| --- | --- | --- | --- | --- | --- | --- |
| Sex *Women* | 49 (75%) | 44 (69%) | 48 (73%) | 0.70 | |  |
| Age (years) | 47.8 (44.8;50.8) | 46.6 (43.9;49.3) | 38.2 (35.3;41.1) | <0.001 | |  |
| Smokers | 8 (12%) | 7 (11%) | 11 (17%) | 0.60 | |  |
| Weight (kg) | 107 [101;114] | 122 [112;131] | 148 [133;163] | <0.001 | |  |
| Waist-hip ratio | 0.88 [0.83;0.97] | 0.88 [0.82;1.01] | 0.87 [0.81;0.95] | 0.63 | |  |
| ALT (U/L) | 38 [26;54] | 31 [24;53] | 31 [20;58] | 0.12 | |  |
| AST (U/L) | 28 [23;35] | 25 [20;36] | 23 [20;31] | 0.10 | |  |
| Creatinine (µmol/L) | 66 [58;73] | 66 [58;78] | 64 [60;74] | 0.87 | |  |
| Total cholesterol (mmol/L) | 4.5 (4.2;4.8) | 4.5 (4.2;4.7) | 4.6 (4.4;4.8) | 0.84 | |  |
| HDL cholesterol (mmol/L) | 1.2 (1.1;1.2) | 1.3 (0.9;1.7) | 1.1 (1.0;1.2) | 0.71 | |  |
| LDL cholesterol (mmol/L) | 2.9 (2.6;3.1) | 3.1 (2.8;3.3) | 3.1 (2.9;3.3) | 0.29 | |  |
| HbA1c (mmol/mol) | 38 [35;42] | 36 [34;41] | 38 [34;43] | 0.46 | |  |
| Total fibrinogen (g/L) | 3.9 (3.7;4.1) | 3.9 (3.7;4.1) | 4.2 (4.0;4.4) | 0.01 | |  |
| Medication *Antihypertensives*  *Antidiabetics*  *Metformin*  *GLP-1 analogs*  *SGLT-2 inhibitors*  *Insulin*  *Others**  *Statins*  *Antidepressants*  *Oral contraceptives* | 31 (48%)  22 (34%)  18 (28%)  7 (11%)  7 (11%)  2 (3%)  1 (2%)  29 (45%)  25 (38%)  5 (8%) | 28 (44%)  13 (20%)  8 (12%)  6 (9%)  1 (2%)  2 (3%)  2 (3%)  18 (28%)  24 (38%)  4 (6%) | 25 (38%)  12 (18%)  11 (17%)  4 (6%)  3 (5%)  2 (3%)  0 (0%)  4 (6%)  18 (27%)  3 (5%) | 0.52  0.08  0.08  0.60  0.07  1.00  0.22  <0.001  0.33  0.76 | |  |

# Data are presented as mean (95% CI), median [IQR], or number (percentages). BMI groups were compared using ANOVA, Kruskal-Wallis test, or Pearson’s chi-squared/Fisher’s exact test as appropriate. BMI, body mass index; ALT, alanine transaminase; AST, aspartate transaminase; HDL, high-density lipoprotein; LDL, low-density lipoprotein; HbA1c, hemoglobin A1c; GLP-1, glucagon-like peptide-1; SGLT-2, sodium-glucose cotransporter-2. *Others include dipeptidyl peptidase (DPP)-IV-inhibitors and sulfonylurea drugs. Previously published by Pedersen et al. [20].

# Table S2. Clinical characteristics of MASLD groups

| Variable | No MASLD (n=55) | MASLD (n=96) | MASH (n=44) | P |
| --- | --- | --- | --- | --- |
| Sex *Women* | 48 (87%) | 60 (62%) | 33 (75%) | 0.004 |
| Age (years) | 40.8 (37.9;43.8) | 45.3 (42.6;47.9) | 45.9 (42.3;49.6) | 0.056 |
| Smokers | 6 (11%) | 14 (15%) | 6 (14%) | 0.88 |
| Weight (kg) | 121 [104;132] | 125 [111;149] | 126 [106;139] | 0.19 |
| BMI (kg/m^2^) | 42.7 (41.3;44.1) | 43.5 (42.0;45.0) | 43.0 (41.3;44.8) | 0.76 |
| Waist-hip ratio | 0.82 [0.78;0.90] | 0.91 [0.83;1.03] | 0.88 [0.85;0.97] | <0.001 |
| ALT (U/L) | 22 [18;31] | 35 [25;49] | 58.0 [32;92] | <0.001 |
| AST (U/L) | 22 [18;25] | 25 [21;33] | 35 [27;57] | <0.001 |
| Creatinine (µmol/L) | 64 [58;72] | 67 [59;76] | 67 [57;79] | 0.48 |
| Total cholesterol (mmol/L) | 4.4 (4.1;4.7) | 4.5 (4.3;4.7) | 4.8 (4.4;5.1) | 0.19 |
| HDL cholesterol (mmol/L) | 1.2 (1.1;1.3) | 1.1 (1.0;1.2) | 1.4 (0.8;2.0) | 0.32 |
| LDL cholesterol (mmol/L) | 3.0 (2.7;3.2) | 3.0 (2.8;3.2) | 3.2 (2.8;3.5) | 0.52 |
| HbA1c (mmol/mol) | 35 [33;38] | 38 [35;43] | 41 [36;52] | <0.001 |
| Total fibrinogen (g/L) | 4.1 (3.9;4.3) | 3.9 (3.8;4.1) | 4.0 (3.8;4.2) | 0.36 |
| Medication *Antihypertensives*  *Antidiabetics*  *Metformin*  *GLP-1 analogs*  *SGLT-2 inhibitors*  *Insulin*  *Others**  *Statins*  *Antidepressants*  *Oral contraceptives* | 18 (33%)  5 (9%)  3 (5%)  2 (4%)  1 (2%)  0 (0%)  0 (0%)  9 (16%)  18 (33%)  6 (11%) | 47 (49%)  27 (28%)  22 (23%)  12 (12%)  9 (9%)  1 (1%)  1 (1%)  28 (29%)  31 (32%)  4 (4%) | 19 (43%)  15 (34%)  12 (27%)  3 (7%)  1 (2%)  5 (11%)  2 (5%)  14 (32%)  18 (41%)  2 (5%) | 0.15  0.004  0.004  0.17  0.10  0.003  0.20  0.14  0.58  0.26 |

Data are presented as mean (95% CI), median [IQR], or number (percentages). MASLD groups were compared using ANOVA, Kruskal-Wallis test, or Pearson’s chi-squared/Fisher’s exact test as appropriate. MASLD, metabolic dysfunction-associated steatotic liver disease; MASH, metabolic dysfunction-associated steatohepatitis; BMI, body mass index; ALT, alanine transaminase; AST, aspartate transaminase; HDL, high-density lipoprotein; LDL, low-density lipoprotein; HbA1c, hemoglobin A1c; GLP-1, glucagon-like peptide-1; SGLT-2, sodium-glucose cotransporter-2. *Others include dipeptidyl peptidase (DPP)-IV-inhibitors and sulfonylurea drugs. Previously published by Pedersen et al. [20].

Table S3. Clinical characteristics of fibrosis groups

| Variable | No fibrosis (n=47) | Mild fibrosis (n=91) | Clinically significant fibrosis (n=57) | P |
| --- | --- | --- | --- | --- |
| Sex *Women* | 43 (91%) | 65 (71%) | 33 (58%) | <0.001 |
| Age (years) | 41.2 (37.6;44.8) | 44.0 (41.5;46.5) | 46.8 (43.5;50.1) | 0.06 |
| Smokers | 8 (17%) | 12 (13%) | 6 (11%) | 0.67 |
| Weight (kg) | 119 [103;130] | 124 [111;142] | 131 [106;141] | 0.16 |
| BMI (kg/m^2^) | 42.9 (41.4;44.5) | 43.2 (41.9;44.4) | 43.3 (41.2;45.4) | 0.96 |
| Waist-hip ratio | 0.83 [0.79;0.89] | 0.88 [0.82;1.00] | 0.93 [0.87;1.03] | <0.001 |
| ALT (U/L) | 21 [17;38] | 33 [24;47] | 47 [29;94] | <0.001 |
| AST (U/L) | 22 [17;26] | 24 [21;30] | 34 [27;57] | <0.001 |
| Creatinine (µmol/L) | 64 [56;70] | 67 [59;78] | 66 [59;78] | 0.18 |
| Total cholesterol (mmol/L) | 4.6 (4.3;5.0) | 4.5 (4.2;4.7) | 4.5 (4.2;4.8) | 0.65 |
| HDL cholesterol (mmol/L) | 1.2 (1.1;1.3) | 1.2 (1.0;1.5) | 1.1 (1.0;1.1) | 0.59 |
| LDL cholesterol (mmol/L) | 3.1 (2.8;3.4) | 3.0 (2.8;3.2) | 3.0 (2.7;3.2) | 0.74 |
| HbA1c (mmol/mol) | 36 [33;40] | 37 [34;41] | 41 [35;49] | 0.005 |
| Total fibrinogen (g/L) | 4.1 (3.8;4.3) | 4.0 (3.8;4.1) | 4.0 (3.8;4.2) | 0.74 |
| Medication *Antihypertensives*  *Antidiabetics*  *Metformin*  *GLP-1 analogs*  *SGLT-2 inhibitors*  *Insulin*  *Others**  *Statins*  *Antidepressants*  *Oral contraceptives* | 11 (23%)  16 (13%)  3 (6%)  3 (6%)  1 (2%)  1 (2%)  0 (0%)  9 (19%)  19 (40%)  5 (11%) | 37 (41%)  19 (21%)  17 (19%)  9 (10%)  5 (5%)  3 (3%)  0 (0%)  19 (21%)  32 (35%)  5 (5%) | 36 (63%)  22 (39%)  17 (30%)  5 (9%)  5 (9%)  2 (4%)  3 (5%)  23 (40%)  16 (28%)  2 (4%) | <0.001  0.007  0.009  0.90  0.40  1.00  0.04  0.02  0.43  0.32 |

Data are presented as mean (95% CI), median [IQR], or number (percentages). Fibrosis groups were compared using ANOVA, Kruskal-Wallis test, or Pearson’s chi-squared/Fisher’s exact test as appropriate. BMI, body mass index; ALT, alanine transaminase; AST, aspartate transami­nase; HDL, high density lipoprotein; LDL, low density lipoprotein; HbA1c, hemoglobin A1c; GLP-1, glucagon-like peptide-1; SGLT-2, sodium-glucose cotransporter-2. *Others include dipeptidyl peptidase (DDP)-IV-inhibitors and sulfonylurea drugs. Previously published by Pedersen et al. [20].

Table S4. Clinical characteristics at baseline and end of study for the intervention (bariatric surgery) and control groups

| Variable | Intervention group (n=35) | | |  | Control group (n=58) | | | |
| --- | --- | --- | --- | --- | --- | --- | --- | --- |
|  | Baseline | 2 years | P |  | Baseline | | 2 years | P |
| Sex  *Women* | 22 (63%) |  |  |  | 43 (74%) |  | |  |
| Age (years) | 42.7 (38.8;46.5) |  |  |  | 45.8 (42.4;49.3) |  | |  |
| Smokers | 5 (14%) |  |  |  | 8 (14%) |  | |  |
| Weight (kg) | 124 [114;152] | 88 [74;102]^$^ | <0.001 |  | 118 [106;133] | 115 [104;131] | | 0.03 |
| BMI (kg/m^2^) | 44.9 (42.3;47.6)^#^ | 31.4 (28.9;34.0)^$^ | <0.001 |  | 41.7 (40.4;43.0) | 40.8 (39.3;42.3) | | 0.03 |
| Waist-hip ratio | 0.91 [0.81;1.03] | 0.88 [0.82;0.96]^$^ | 0.001 |  | 0.88 [0.84;0.95] | 0.89 [0.84;0.95] | | 0.91 |
| SAF score^a^  *No MASLD*  *MASLD*  *MASH* | 13 (37%)  16 (46%)  6 (17%) | 27 (96%)^$^  1 (4%)^$^  0 (0%)^$^ | <0.001 |  | 12 (21%)  27 (47%)  19 (33%) | 13 (24%)  26 (48%)  15 (28%) | | 0.51 |
| Fibrosis grade^a^  *No fibrosis*  *Mild fibrosis*  *Clinically significant fibrosis* | 12 (34%)  15 (43%)  8 (23%) | 11 (31%)  14 (40%)  10 (29%) | 0.74 |  | 16 (28%)  31 (53%)  11 (19%) | 12 (21%)  23 (40%)  23 (40%) | | 0.001 |
| ALT (U/L) | 27 [21;44] | 24 [17;31]^$^ | 0.001 |  | 34 [21;52] | 32 [22;46] | | 0.41 |
| AST (U/L) | 23 [20;30] | 22 [19;26]^$^ | 0.21 |  | 25 [21;33] | 27 [20;33] | | 0.68 |
| Creatinine (µmol/L) | 68 [60;79] | 61 [53;70] | <0.001 |  | 69 [63;78] | 66 [57;74] | | 0.003 |
| Total cholesterol (mmol/L) | 4.3 (3.9;4.7) | 4.1 (3.7;4.4) | 0.19 |  | 4.8 (4.5;5.1) | 5.8 (4.1;7.5) | | 0.24 |
| HDL cholesterol (mmol/L) | 1.1 (1.0;1.3) | 1.5 (1.3;1.6) | <0.001 |  | 1.2 (1.1;1.2) | 1.4 (1.0;1.8) | | 0.18 |
| LDL cholesterol (mmol/L) | 2.8 (2.5;3.2) | 2.4 (2.1;2.7) | 0.01 |  | 3.2 (2.9;3.5) | 3.8 (2.7;4.9) | | 0.32 |
| HbA1c (mmol/mol) | 36 [34;42] | 32 [28;36]^$^ | <0.001 |  | 38 [34;45] | 37 [33;46] | | 0.42 |
| Total fibrinogen (g/L) | 4.1 (3.9;4.4) | 3.4 (3.1;3.6) | <0.001 |  | 3.9 (3.7;4.1) | 3.9 (3.7;4.1) | | 0.83 |
| Medication  *Antihypertensives*  *Antidiabetics*  *Metformin*  *GLP-1 analogs*  *SGLT-2 inhibitors*  *Insulin*  *Others**  *Statins*  *Antidepressants*  *Oral contraceptives* | 15 (43%)  7 (20%)  4 (11%)  1 (3%)  2 (6%)  3 (9%)  1 (3%)  7 (20%)  12 (34%)  3 (9%) | 8 (23%)^$^  3 (9%)^$^  1 (3%)^$^  2 (6%)  0 (0%)^$^  0 (0%)  0 (0%)  3 (9%)^$^  12 (34%)  1 (3%) | 0.04  0.13  0.25  1.00  0.50  0.25  1.00  0.22  1.00  0.63 |  | 18 (31%)  12 (21%)  10 (17%)  6 (10%)  4 (7%)  2 (3%)  1 (2%)  15 (26%)  20 (34%)  4 (7%) | 25 (43%)  18 (31%)  15 (26%)  10 (17%)  6 (10%)  4 (7%)  0 (0%)  17 (29%)  22 (38%)  2 (3%) | | 0.02  0.11  0.23  0.39  0.63  0.63  1.00  0.73  0.73  0.50 |

# Data are presented as mean (95% CI), median [IQR], or number (percentages). Groups at baseline were compared using unpaired t-test, Mann-Whitney U-test, or Pearson’s chi-squared/Fisher’s exact test as appropriate. Within-group comparisons were performed using a paired t-test, Wilcoxon signed-rank test, or McNemar’s/Stuart-Maxwell test as appropriate. Linear regression analysis was used to assess associations between surgery (exposure) and clinical characteristics (outcome) after the follow-up period, with adjustment for baseline values. BMI, body mass index; SAF, steatosis, activity, and fibrosis; MASLD, metabolic dysfunction-associated steatotic liver disease; MASH, metabolic dysfunction-associated steatohepatitis; ALT, alanine transaminase; AST, aspartate transaminase; HDL, high-density lipoprotein; LDL, low-density lipoprotein; HbA1c, hemoglobin A1c; GLP-1, glucagon-like peptide-1; SGLT-2, sodium-glucose cotransporter-2. *Others include dipeptidyl peptidase (DDP)-IV-inhibitors and sulfonylurea drugs. ^#^Significantly higher than the control group at baseline (p=0.02). ^$^Significantly lower than control group at end of study (weight, p<0.001; BMI, p<0.001; waist-hip ratio, p=0.002; SAF score, p<0.001; ALT, p=0.002; AST, p=0.03; HbA1c, p=0.03; antihypertensives, p=0.048; antidiabetics, p=0.01, statins, p=0.02). ^a^Based on liver biopsies from 33 individuals in the intervention group and 55 individuals in the control group. Previously published by Pedersen et al. [20].


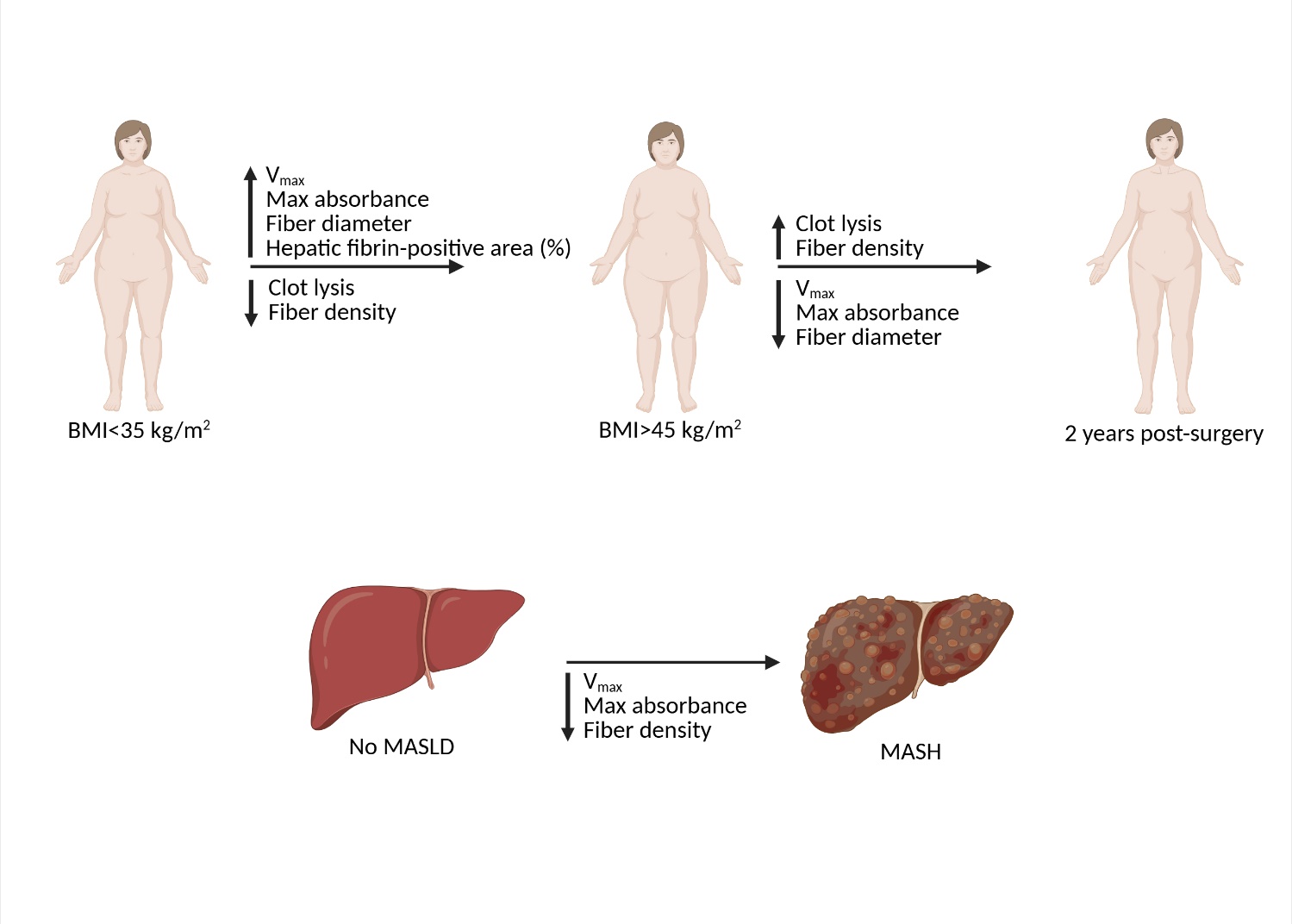


**Figure S5.** Summary of results presented in the study. BMI, body mass index; V_max_, maximal turbidity increment; MASLD, metabolic dysfunction-associated steatotic liver disease; MASH, metabolic dysfunction-associated steatohepatitis. Created in BioRender. Lauridsen, M. (2025) <https://BioRender.com/pzjxyac>
